# Hydrophobia

**Published:** 1853-11

**Authors:** 


*Hydrophobia.*

In chapter x., p. 150, of Mr. E. E. Crowe's "The Greek and the Turk, or Prospects, &c., in the Levant," I find the following: "A physician of that city (Athens) has discovered in the stems of asparagus what he considers a certain specific against hydrophobia, and asparagus is, fortunately, to be had at those seasons when hydrophobia declares itself. The physician has had frequent occasion to employ his specific, and always with success."

From the "Materia Medica" it appears that a decoction is made from asparagus as follows:—"Take of the root of asparagus, one ounce; water, two pounds; boil and strain. It is stimulant, and reputed diuretic. It is taken as a common drink in dropsies."

Query what would be the strength of such a medicine in a form stronger than a decoction? Mr. Cowe does state the amount of the dose to be given, and how often daily, &c. W. H.

Oriental Club, September, 1853.

